# Adult Patients with Congenital Adrenal Hyperplasia Have Elevated Blood Pressure but Otherwise a Normal Cardiovascular Risk Profile

**DOI:** 10.1371/journal.pone.0024204

**Published:** 2011-09-01

**Authors:** Christiaan F. Mooij, Jeanne Margot Kroese, Fred C. G. J. Sweep, Ad R. M. M. Hermus, Cees J. Tack

**Affiliations:** 1 Department of Endocrinology, Radboud University Nijmegen Medical Centre, Nijmegen, The Netherlands; 2 Department of Paediatric Endocrinology, Radboud University Nijmegen Medical Centre, Nijmegen, The Netherlands; 3 Department of Internal Medicine, Radboud University Nijmegen Medical Centre, Nijmegen, The Netherlands; 4 Department of Laboratory Medicine, Radboud University Nijmegen Medical Centre, Nijmegen, The Netherlands; Universidad Peruana Cayetano Heredia, Peru

## Abstract

**Objective:**

Treatment with glucocorticoids and mineralocorticoids has changed congenital adrenal hyperplasia (CAH) from a fatal to a chronic lifelong disease. Long-term treatment, in particular the chronic (over-)treatment with glucocorticoids, may have an adverse effect on the cardiovascular risk profile in adult CAH patients. The objective of this study was to evaluate the cardiovascular risk profile of adult CAH patients.

**Design:**

Case-control study.

**Patients and Measurements:**

In this case-control study the cardiovascular risk profile of 27 adult CAH patients and 27 controls, matched for age, sex and body mass index was evaluated by measuring ambulatory 24-hour blood pressure, insulin sensitivity (HOMA-IR), lipid profiles, albuminuria and circulating cardiovascular risk markers (PAI-1, tPA, uPA, tPA/PAI-1 complex, hsCRP, adiponectin, IL-6, IL-18 and leptin).

**Results:**

24-Hour systolic (126.3 mmHg±15.5 *vs* 124.8 mmHg±15.1 in controls, *P* = 0.019) and diastolic (76.4 mmHg±12.7 *vs* 73.5 mmHg±12.4 in controls, *P*<0.001) blood pressure was significantly elevated in CAH patients compared to the control population. CAH patients had higher HDL cholesterol levels (*P*<0.01), lower hsCRP levels (*P* = 0.03) and there was a trend toward elevated adiponectin levels compared to controls. Other cardiovascular risk factors were similar in both groups.

**Conclusion:**

Adult CAH patients have higher ambulatory blood pressure compared to healthy matched controls. Other cardiovascular risk markers did not differ, while HDL-cholesterol, hsCRP and adiponectin levels tended to be more favorable.

## Introduction

Congenital adrenal hyperplasia (CAH) is an autosomal recessive disorder of adrenal steroidogenesis. In 95% of cases it is caused by 21-hydroxylase deficiency.[1] Deficiency of 21-hydroxylase results in impaired adrenal synthesis of cortisol and often also of aldosterone leading to increased secretion of ACTH by the pituitary gland, adrenal hyperplasia, and excessive production of adrenal androgens. Current treatment of CAH consists of administration of glucocorticoids and, if necessary, of mineralocorticoids to prevent adrenal crises and to suppress the abnormal secretion of adrenal androgens. Patients with CAH are at risk of developing signs and symptoms of Cushing's syndrome, as the therapeutic range of treatment with glucocorticoids is narrow and slightly supraphysiological doses of glucocorticoids are needed.[1], [2] Cushing's syndrome is associated with insulin resistance and cardiovascular morbidity.[3] Therefore, patients with CAH may develop an adverse cardiovascular risk profile, which is of increasing clinical importance as nowadays nearly all CAH patients reach adulthood.

A recent Swedish population study described an increased risk ratio for all-cause mortality of 2.19 (confidence interval 1.91–2.51) for men and 2.86 (confidence interval 2.54–3.20) for women treated with glucocorticoids in Addison's disease. [4] The excess mortality in both males and females was mainly attributable to cardiovascular disease (risk ratio (RR) for cardiovascular death in men 1.97 (confidence interval 1.61–2.39); RR in women 2.31 (confidence interval 1.94–2.74)). The increased cardiovascular mortality in Addison's patients is most likely caused by excess glucocorticoid exposure. CAH patients are commonly treated with significantly higher doses of glucocorticoids than Addison's patients, already starting directly after birth. Therefore, evaluating the cardiovascular risk profile in CAH patients is of importance, with the oldest CAH patients now being in their sixties.

So far, few studies have focused on cardiovascular risk in CAH patients. An elevated body mass, an increased fat mass and insulin resistance have been described in adult and pediatric CAH patients.[5] Blood pressure has been studied mainly in pediatric and young adult CAH patients and showed a tendency towards high blood pressure in some, but not all, studies.[5] Lipid profiles in adult CAH patients did not show unfavorable changes.[5]

We hypothesized that adult CAH patients are at risk to develop an unfavorable cardiovascular risk profile due to lifelong treatment with glucocorticoids. As described above, results of prior studies concerning the cardiovascular risk profile in CAH patients are inconclusive. Furthermore, most studies were performed in small patient groups, or mainly in pediatric patients or without a proper control group. As adult CAH patients tend to become obese, especially proper matching for body mass is warranted. More recent markers for cardiovascular risk, like plasminogen activator inhibitor type 1 (PAI-1), tissue-type plasminogen activator (tPA), urokinase-type plasminogen activator (uPA), tPA/PAI-1 complex and high sensitive CRP (hsCRP), have not been studied before in CAH patients. Therefore, the aim of this study was to evaluate insulin sensitivity, blood pressure, albuminuria, lipid profile, and other circulating cardiovascular risk markers in adult CAH patients and to compare to the cardiovascular risk profile of carefully matched control subjects.

## Materials and Methods

### Patients

Adult patients with proven congenital adrenal hyperplasia were included in this study. Inclusion criteria for patients were biochemically and genetically proven CAH and stable glucocorticoid and mineralocorticoid therapy for 3 months. For each individual patient a healthy, age-, sex- and body mass index (BMI)-matched control was recruited by advertisements. Inclusion criteria for control subjects were an unremarkable medical history and at present no evidence of disease, no medication (except for oral contraceptives) and Caucasian race. Exclusion criteria for patients and control subjects were age <18 years and inability to give informed consent. All patients were fully informed about the aim and design of the study and all the methods involved. They consented with the study protocol according to the recommendations of the medical ethical committee of the Radboud University Nijmegen Medical Centre.

### Methods

Each participant visited the hospital on two consecutive days. On the first day, participants visited the hospital in the morning after abstinence of caffeine containing substances and an overnight (10-hour) fast. All participants received a physical examination including anthropometric measurements. Blood was drawn to assess fasting glucose, insulin, HDL cholesterol, LDL cholesterol, total cholesterol, triglycerides, and several circulating cardiovascular risk markers, like PAI-1, tPA, uPA, tPA/PAI-1 complex, hsCRP, adiponectin, IL-6, IL-18 and leptin. Plasma concentrations of total adiponectin, IL-6 and IL-18, hsCRP and leptin were determined using ELISAs (R&D Systems, Minneapolis, MN). ELISAs, developed by our department, were used for assessment of components of the plasminogen activation system (uPA, tPA and PAI-1) and its complexes (tPA:PAI-1).[6], [7] PAI-1/tPA ratios were calculated. Insulin resistance (IR) was estimated using the homeostasis model assessment (HOMA) method [IR = insulin (µmol/ml)×glucose (mmol/l)/22.5].[8] Office blood pressure was measured using a Dinamap Vital Signs Monitor. Blood pressure was measured twice supine and once in upright position. Mean supine office blood pressure was calculated. Subsequently, ambulatory blood pressure was monitored for 24 hours (SpaceLabs model 90207). On the second day, participants returned to the hospital for disconnection of the ambulatory blood pressure monitoring device.

### Statistics

For calculations and statistical analyses, the SPSS personal computer software package was used and *P*<0.05 was considered statistically significant. Abnormally distributed data were log-transformed. Differences between CAH patients and controls were statistically tested using unpaired Student's *t*-test or the Mann-Whitney U-test, as appropriate. Results are expressed as mean ± SEM, unless otherwise indicated.

## Results

A total of 27 patients were included in this study. 20 patients had the salt-wasting type of CAH, 6 patients were simple virilizers and 1 patient had a non-classic type of CAH. The control group was well matched for age, sex and BMI. Key parameters of the selected population were similar to the whole population of adult CAH patients treated in our medical centre. At the time of the investigation twenty-three patients were treated with hydrocortisone as the only glucocorticoid. One patient was treated with dexamethasone only, one patient was treated with cortisone acetate only and two patients received a combination of dexamethasone and hydrocortisone. Glucocorticoid doses were converted into hydrocortisone equivalents using anti-inflammatory equivalents (hydrocortisone 30 mg = cortisone acetate 37.5 mg = dexamethasone 0.75 mg). Hydrocortisone equivalents are also presented as mg/m^2^. Five patients had plasma renin levels <5 mU/l, consistent with overtreatment. Characteristics of our study population, including current daily hydrocortisone and fludrocortisone dosage, are shown in table 1.

**Table 1:** Characteristics of the study population.

	Patients (n = 27)	Controls (n = 27)
**Gender (M/F)**	12/15	12/15
**Age (years)**	30.2±8.0	32.5±11.7
**Height (m)**	1.67±0.10	1.75±0.09#
**Weight (kg)**	75.4±13.1	83.3±14.5#
**BMI (kg/m^2^)**	27.2±4.6	27.3±4.8
**Waist circumference (cm)**	86.9±11.5	88.3±12.0
**Hip circumference (cm)**	101.6±9.7	103.9±9.4
**Waist/hip ratio**	0.86±0.08	0.85±0.08
**Office systolic blood pressure (mmHg)**		
Supine	133±12	133±12
Upright	128±13	131±12
**Office diastolic blood pressure (mmHg)**		
Supine	83±10	80±10
Upright	91±10	90±10
**Office heart rate (bpm)**		
Supine	67±13	66±11
Upright	78±14	79±14
**Daily hydrocortisone dosage (mg)**	23.4±8.0	-
**Daily hydrocortisone dosage (mg/m^2^)** *	12.6±4.48	-
**Daily fludrocortisone dosage (mg)** **	0.11±0.06	-
**Plasma renin level (mE/l)**	34.9±69.9	-

Mean values±1 SD are given.

M = male; F = female; bpm = beats per minute,

# *P*<0.05,

*Glucocorticoid doses were converted into hydrocortisone equivalents using anti-inflammatory equivalents,

**25 patients were treated with fludrocortisone.

### Blood pressure profiles

Office blood pressure measurements during hospital visit showed no significant differences in supine and upright systolic and diastolic blood pressure between CAH patients and controls. Supine and upright heart rates during hospital visit were not different in CAH patients compared to controls (table 1).

**Figure 1:**
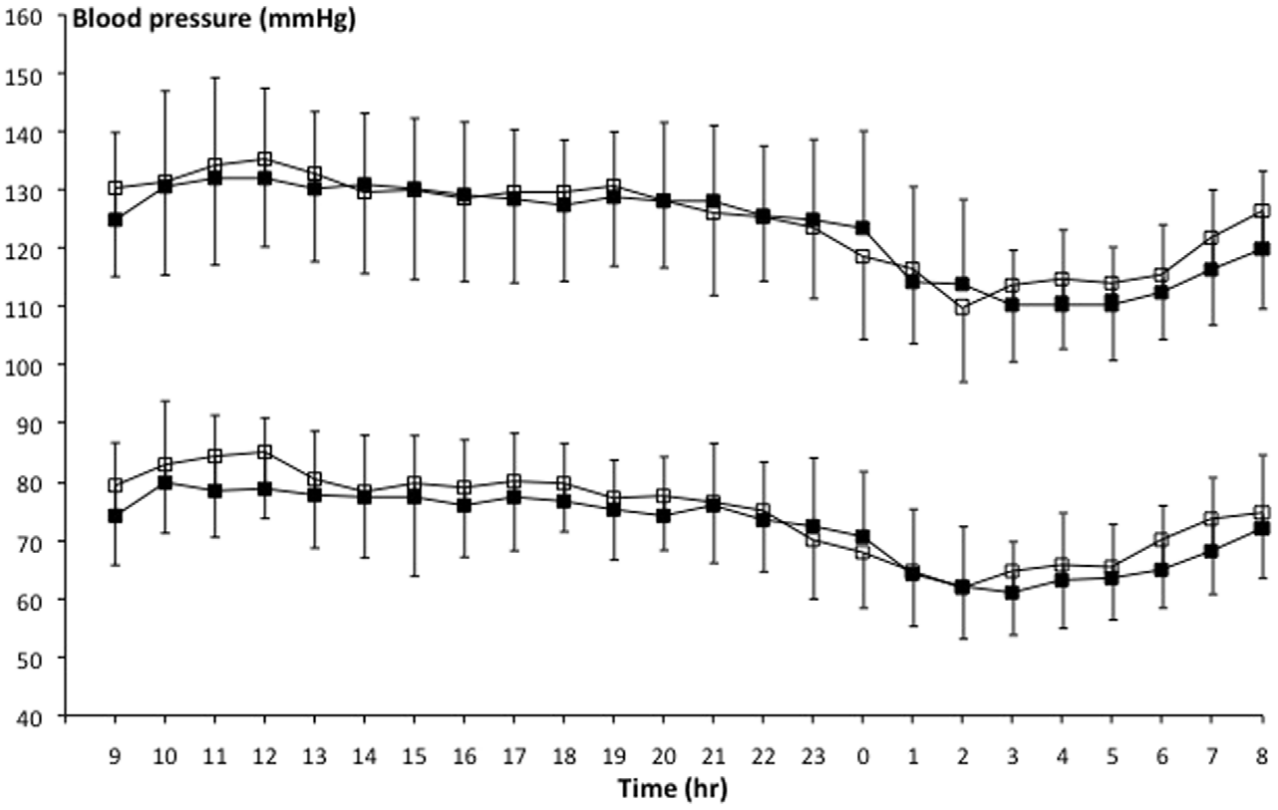
Mean 24-hour ambulatory blood pressure measurements (±1 SD) in CAH patients (open squares) and matched controls (closed squares).

Mean 24-hour systolic (mean ± SD, 126.3±15.5 mmHg *vs* 124.8±15.1 mmHg, *P* = 0.019) and diastolic (76.4±12.7 mmHg *vs* 73.5±12.4 mmHg, *P*<0.001) blood pressure was significantly elevated in CAH patients compared to the control population. Mean arterial pressure (MAP, 92.7±12.9 mmHg *vs* 90.0±12.3 mmHg in controls, *P*<0.001) and heart rate (77.1 bpm±17.9 *vs* 74.2 bpm±15.5 in controls, *P*<0.001) also showed significantly higher levels in CAH patients than in controls. Measured blood pressure values and differences between day- and nighttime are shown in figure 1.

When analyzed separately for day and night, systolic blood pressure was not significantly elevated during the day (08.00 h to 22.00 h) nor during the night (22.00 h to 08.00 h), but diastolic blood pressure was elevated both during day- (*P*<0.001) and nighttime (*P* = 0.001) in CAH patients compared to controls. CAH patients thus had a similar blood pressure dip during the night as compared to controls.

### Urinary cardiovascular risk markers

Albumin excretion was comparable in CAH patients and controls (data not shown).

### Lipid profile

HDL cholesterol concentration was higher in CAH patients compared to controls. No significant differences in total cholesterol, LDL cholesterol and triglycerides between CAH patients and controls were observed (Table 2).

**Table 2:** Lipid profile in adult CAH patients and healthy, matched controls.

	CAH patients (n = 27)	Controls (n = 27)	*P*-value
**Total cholesterol (mmol/l)**	4.6±1.0	4.2±1.0	0.22
**Triglycerides (mmol/l)**	1.0±0.6	1.0±0.6	0.93
**HDL cholesterol (mmol/l)**	1.4±0.3	1.1±0.2	<0.01
**LDL cholesterol (mmol/l)**	2.8±0.7	2.7±0.9	0.87

Mean values±1 SD are given.

### Insulin sensitivity

Fasting plasma glucose levels did not differ between CAH patients and controls (4.6±0.6 vs 4.8±0.6 mmol/L, *P* = not significant). Insulin levels also were comparable in CAH patients and controls (9.4±4.6 vs 10.1±4.8 pmol/L, *P* = not significant), as was calculated HOMA-IR (2.0±1.2 vs 2.2±1.0, *P* = not significant).

### Circulating cardiovascular risk markers

A trend toward elevated adiponectin levels in CAH patients compared to controls was observed (Table 3). Significantly lower hsCRP levels were found in CAH patients compared to controls (*P* = 0.03). PAI-1, uPA, tPA, tPA/PA1-complex, PAI-1/tPA ratio, IL-6, IL-18 and leptin levels were not significantly different in CAH patients and controls (Table 2).

**Table 3:** Circulating cardiovascular risk markers in adult CAH patients and healthy, matched controls.

	CAH patients (n = 27)	Controls (n = 27)	*P*-value
**IL-18 (pg/mL)**	57±24	53±26	0.56
**IL-6 (pg/mL)**	1.31±1.50	1.37±1.26	0.89
**Adiponectin(ug/mL)**	4.17±3.03	2.93±1.33	0.06
**Leptin (ng/mL)**	24±20 (n = 26)	24±18	0.92
**hsCRP (mg/L)** *	1.06±1.49	1.79±1.83	0.03
**uPA (ng/mL)**	0.99±0.52	0.92±0.54	0.63
**PAI-1 (ng/mL)**	55±29	51±19	0.51
**tPA (ng/mL)**	4.86±2.13	6.01±3.74	0.17
**tPA-PAI-1 complex (ng/mL)**	6.95±3.67	9.11±5.52	0.10
**PAI-1/tPA ratio**	12.53±7.72	10.19±4.23	0.17

Mean values±1 SD are given.

*As hsCRP values were not distributed normally, we have calculated log(CRP) values.

### Clinical characteristics

Average BMI was high in CAH patients (27.2±4.6 kg/m^2^). Waist and hip circumference and waist/hip ratio were not different in CAH patients compared to controls (data shown in table 1). It was not possible to evaluate BMI as an individual cardiovascular risk marker because controls were matched for BMI in our study.

## Discussion

The present study shows that the cardiovascular risk profile of adult CAH patients is relatively unaffected compared to a carefully BMI, sex and age matched control group; only slightly elevated 24-hour blood pressure levels were found.

Our finding of an elevated 24-hour blood pressure profile in adult CAH patients was not reported in earlier studies in adult CAH patients.[9], [10] However, studies in young adult and pediatric CAH patients did show a tendency towards hypertension.[5] Elevated blood pressure in CAH patients may be caused both by glucocorticoid and mineralocorticoid therapy, as glucocorticoid as well as mineralocorticoid excess is known to result in high blood pressure.[11], [12] As 24-hour blood pressure is only slightly elevated it is hard to predict the clinical importance of this finding for the individual patient. Therefore, it is uncertain if the elevation in 24-hour blood pressure profile found in our population will lead to an increased cardiovascular mortality. The increase was found in ambulatory blood pressure, not in office blood pressure, suggesting that a given office blood pressure in a CAH patient may reflect slightly higher 24 h hypertensive burden and thus more risk compared to control subjects.

The finding of a tendency toward elevated adiponectin levels in adult CAH patients is in line with the study by Völkl et al. who reported significantly elevated adiponectin levels in 51 children and adolescents with CAH.[13] Adiponectin has insulin sensitizing and anti-inflammatory properties and high adiponectin levels are associated with lower risk of myocardial infarction and a reduced risk of type 2 diabetes.[14], [15] Adiponectin levels are known to be decreased in obesity, insulin resistance and type 2 diabetes,[16] and administration of glucocorticoids and androgens have been shown to decrease adiponectin levels.[17], [18] The higher adiponectin level found in CAH patients treated with glucocorticoids is thus somewhat surprising. With respect to their cardiovascular risk profile, elevated adiponectin levels might have a beneficial effect concerning the development of type 2 diabetes and myocardial infarction.

In contrast to earlier studies,[5] including one of our own,[19] our adult CAH patients were not insulin resistant when evaluated by the HOMA-IR method. The probable explanation for this finding is the careful matching in BMI. Alternatively, the HOMA method may not have been sensitive enough to detect differences in our population. The lipid profile of adult CAH-patients showed no unfavorable changes. This finding is in line with earlier studies evaluating lipid profiles.[5] In the present study we found significantly elevated HDL-cholesterol levels; Falhammer *et al* showed a tendency towards higher HDL-cholesterol in female CAH patients 30 years of age or older.[9]

To our knowledge hsCRP levels have not been studied before in CAH patients. The trend towards lower hsCRP levels in CAH patients may be explained by the chronic treatment with glucocorticoids. It is well known that glucocorticoids have anti-inflammatory effects, but data on the direct effect of glucocorticoid treatment on CRP levels are lacking.[20]

Most studies evaluating cardiovascular risk factors in CAH patients have focussed on one or several risk factors. Recently, the CaHASE study evaluated the health status, including cardiovascular and metabolic risk, in a large British cohort of 199 adult CAH patients due to 21-hydroxylase deficiency. [21] Results were compared to Health Survey for England data. Similar to our study they showed elevated diastolic but not systolic blood pressure. Differences in blood pressure results between our study and the CaHASE study may be explained by the fact that in the CaHASE study no 24-hour ambulatory blood pressure measurement was performed and that the results were compared to reference values of the general population. Furthermore they report high frequencies of obesity (41%), hypercholesterolemia (46%), insulin resistance (29%) and osteopenia (40%) compared to reference values. As data in the CaHASE study are only compared to age- and sex-matched reference data it is hard to compare their results with those obtained in our study, using an age-, sex- and BMI-matched cohort, as increased BMI plays an important role in both cardiovascular and metabolic risk. Falhammar et al. recently evaluated cardiovascular and metabolic risk in adult male CAH patients. [22] Their findings are partly in agreement with ours: increased fat mass and similar lipid profiles, but no increased blood pressure, which may be due to the small (sub) sample sets.

Our study had limitations. We have used the HOMA-IR method to evaluate insulin sensitivity instead of the gold standard, the euglycemic clamp. Furthermore, the fact that we have chosen to use a BMI-matched control group, precludes us to evaluate the role of obesity as a cardiovascular risk factor in CAH patients. This may be relevant as the CAH group was on average overweight. Evaluation of the cardiovascular risk profile depends on the evaluated markers. In this study we did not evaluate endothelial dysfunction, endothelial vasodilative capacity and the endothelial response to glucocorticoid and mineralocorticoids. As we did not control for the effects of environmental and physiological factors like stress and health behaviour we could not evaluate the role of these factors on blood pressure levels and other cardiovascular risk factors.

In summary, this study shows that adult CAH patients have an elevated ambulatory blood pressure, but otherwise a normal cardiovascular risk profile compared to healthy BMI, age and sex matched controls. One may speculate that elevated adiponectin and HDL-cholesterol and decreased hsCRP levels may even have some protective effect on the development of cardiovascular disease in these patients.
